# Mediators of the association between parental education and breakfast consumption among adolescents : the ESSENS study

**DOI:** 10.1186/s12887-017-0811-2

**Published:** 2017-02-23

**Authors:** Mekdes K. Gebremariam, Sigrun Henjum, Elisabeth Hurum, Jorunn Utne, Laura Terragni, Liv Elin Torheim

**Affiliations:** Department of Nursing and Health Promotion, Faculty of Health Sciences, Oslo and Akershus University College of Applied Sciences, P.O. Box 4, Olavs Plass Street, Oslo, 0130 Norway

**Keywords:** Breakfast, Correlates, Socioeconomic differences, Mediators, Adolescents

## Abstract

**Background:**

Regular breakfast consumption has several health benefits. However, breakfast skipping is common among adolescents, in particular among those with a low socioeconomic background. The aims of the study were to explore individual and home environmental correlates of breakfast consumption, and to assess their potential mediating role in the association between parental education and breakfast consumption.

**Methods:**

A cross-sectional study including 706 adolescents with a mean age of 13.6 (SD = 0.3) was conducted between October and December 2016. Data were collected at school through an online questionnaire. Regression analyses were used to explore whether parental modelling, parental co-participation in breakfast consumption, parental rules, the availability of breakfast foods at home and screen time were associated with breakfast consumption. Mediation analyses were conducted to assess whether these factors mediated the association between parental education and breakfast consumption.

**Results:**

Breakfast consumption was significantly positively associated with parental education (OR = 1.97 (95% CI 1.43–2.72)). A higher parental modelling (OR = 2.17 (95% CI 1.70–2.79)), a higher parental co-participation in breakfast consumption (OR = 1.37 (95% CI 1.26, 1.49)), higher parental rules (OR = 1.36 (95% CI 1.21, 1.53)) and a higher availability of breakfast foods at home (OR = 2.21 (95% CI 1.65, 2.97)) were associated with higher odds of being a daily breakfast consumer. Higher levels of screen time (hrs/day) were associated with lower odds of being a daily breakfast consumer (OR = 0.85 (95% CI 0.79, 0.91). Parental modelling (*B* = 0.254 (95% CI 0.149, 0.358)) and the availability of breakfast foods at home (*B* = 0.124 (95% CI 0.033, 0.214)) were significantly positively related to parental education, whereas screen time (hrs/day) (*B* = −1.134 (95% CI −1.511, −0.758)) was significantly inversely related to parental education. Parental modelling, the availability of breakfast foods at home and screen time were found to mediate parental educational differences in breakfast consumption.

**Conclusions:**

Increasing the availability of breakfast food, improving parental modelling of breakfast consumption and targeting screen time might be promising strategies to reduce parental educational differences in breakfast consumption.

**Electronic supplementary material:**

The online version of this article (doi:10.1186/s12887-017-0811-2) contains supplementary material, which is available to authorized users.

## Background

A high prevalence of breakfast skipping has been documented among adolescents in Norway, with only 63% of adolescents consuming breakfast daily [1]. The same was true for other countries included in the same study, with rates of daily breakfast consumption ranging from 40% in Slovenia to 74% in the Netherlands [1]. This is of great concern as regular breakfast consumption has several health benefits. These include a lower body mass index and lower odds of being overweight [2, 3], a better academic performance [4], and a generally better dietary quality [5–7]. As eating behavior established in adolescence tends to track into adulthood [8, 9], promoting adolescent breakfast consumption is particularly important. In this regard, knowledge about the factors influencing breakfast consumption is vital. Factors found to be related to breakfast consumption in youth include attitudes [10, 11], self-efficacy [10], parental modelling [12–15], parental rules [12, 15], eating breakfast with parents [12, 15, 16] and availability of breakfast foods [12, 15]. As screen time is found to be positively associated with later sleep time [17], it can potentially lead to breakfast skipping among those who wake up late after late sleep. Only a few studies have however looked at the association between breakfast consumption and sedentary behavior including screen-based sedentary behaviors and yielded inverse [18, 19] as well as mixed [20] associations. Systematic reviews indicate that it is difficult to draw firm conclusions about correlates of breakfast consumption, as few studies have addressed the same correlates [13, 14]. The present study will help address this gap by looking at multiple correlates of breakfast consumption among adolescents.

In addition, breakfast consumption has been found to be positively related to socioeconomic position (SEP) in several studies, irrespective of the indicator of SEP used [1, 18, 21–25]. The identification of these socioeconomic differences is important; however exploring modifiable factors explaining these differences is even more relevant from a public health perspective. Nevertheless, studies exploring mediators of socioeconomic differences in breakfast consumption are very scarce. One study found that attitude towards breakfast consumption was a mediator of the association between deprivation and breakfast skipping among 9–11 year-olds [11]. Another study conducted among 11 year-old Dutch children found that parental breakfast consumption mediated the association between maternal education and child breakfast consumption. However, no other potential mediators were taken into consideration [25]. Studies exploring mediators of socioeconomic differences in breakfast consumption and using a broader range of mediators are therefore needed to address this gap in the literature. Such studies will help design effective interventions targeting socioeconomic differences in breakfast consumption.

Against this background, the aim of the present study was to explore correlates of breakfast consumption (parental modelling, parental rules, availability of breakfast foods at home, parental co-participation (eating breakfast with parents) and screen time), and to explore their mediating role in the association between parental education and breakfast consumption.

## Methods

### Design and sample

The participants in this study were pupils from eleven secondary schools participating in the Environmental determinantS of dietary behaviorS among adolescENtS (ESSENS) cross-sectional study. All twelve secondary schools in the Øvre Romerike region located in the Eastern part of Norway were invited to participate in the study, and eleven accepted the invitation. In total, 1163 adolescents in the eighth grade (average age of 13–14 years) were invited to participate in this study and a total of 781 (67%) received parental consent for participation. A total of 742 adolescents (64% of those invited and 95% of those with parental consent) participated in the study. Data collection was conducted between October and December 2015.

### Data collection and measures

A web-based questionnaire was used to collect data from the adolescents, using the LimeSurvey data collection tool. The questionnaires were filled in at school, and took approximately 30–45 min to complete. Research group members were present during data collection to assist participants. The questionnaire contained questions about selected dietary behaviors, physical activity and selected sedentary behaviors, as well as selected correlates of these behaviors. Only five of the questions were mandatory. The questionnaire was pre-tested for clarity and length among a group of adolescents (*n* = 23) of the same age as the study participants, prior to the main study. The questionnaire was subsequently shortened and some questions were rephrased.

### Measures

#### Outcome measure: breakfast consumption

Adolescents’ breakfast consumption was assessed using two questions asking the adolescents on how many schooldays and how many weekend days per week they normally ate breakfast. The answers to these two questions were summed up to create a weekly frequency variable. Then, the adolescents were divided into two groups: those who were daily breakfast consumers and those skipping breakfast at least once a week, as has been used repeatedly in the literature [1, 18, 24]. Breakfast consumption was categorized because the continuous variable was highly skewed and because the recommendation for breakfast intake is not to skip breakfast, therefore allowing us to compare those who meet recommendations vs. those who do not. This question has shown evidence of moderate test-retest reliability (% agreement of 83 and 81% respectively for weekday and weekend measures) and moderate construct validity (% agreement of 80 and 87% respectively for weekday and weekend measures) among 10–12 year old European children [26].

#### Correlates

Perceived parental co-participation was assessed by asking how often the adolescents ate breakfast with their parents (5 answer categories ranging from never to always). Perceived parental modelling of breakfast consumption was assessed using a question asking the adolescents how often their parents ate breakfast (5 answer categories ranging from never to always). Perceived parental rules were assessed by asking the adolescents whether their parents had rules regarding breakfast consumption (5 answer categories ranging from completely agree to completely disagree). The adolescents were also asked whether there usually were breakfast foods (e.g. bread, cereals, milk) present in the home (7 answer categories ranging from never to every day). The questions were adopted from a previously validated questionnaire among European 11 year-olds [26]. For the question assessing parental co-participation, ICC for test-retest reliability was 0.74 and ICC for construct validity was 0.60. The respective ICC values for the question assessing parental modelling were 0.71 and 0.69. For the question assessing parental rules, percentage agreement values for test-retest reliability and construct validity were 79 and 67% respectively; the response options were however modified in the present study. For the question assessing the availability of breakfast food, percentage agreement values for test-retest reliability and construct validity were 75 and 80% respectively [26].

Time spent watching TV during leisure time (including video, DVDs and films on computer, telephone or ipad) was assessed separately for weekdays and weekend days with nine answer categories ranging from “no viewing” to “4 h or more per day”. Time spent on online activities (e.g. chatting, internet surfing, facebook, instagram) during leisure-time was assessed in a similar manner. A third question was used to assess electronic game use (computer game, playstation, games on ipad and games on mobile phone). Weekly total screen time scores were calculated by summing hours reported for an average weekday (multiplied by five) and average weekend day (multiplied by 2) and adding the three screen-based activities together. The screen time measures were adopted and modified (to reflect recent patterns in screen-based activities) from previous measures with evidence of moderate construct validity [26] and moderate test-retest reliability [26, 27]. The questions used in this study are included in Additional file 1.

### Sociodemographic correlates

Two questions assessing parental education were included on the parental informed consent form for the adolescent. The questions assessed the education of guardian 1 and guardian 2 (could be mother, father, stepmother, stepfather, male guardian, female guardian). Parental education was categorized into: low (12 years of education or less, which corresponded to secondary education or lower) and high (13 years of education and more, which corresponded to university or college attendance). Educational status of the parent with the longest education or else the one available was used in the analyses.

Participants were divided into either ethnic Norwegian or ethnic minority. Ethnic minorities were defined as those having both parents born in a country other than Norway [28].

#### Statistical analyses

Since schools were the unit of measurement in this study, we checked for clustering effect through the Linear Mixed Model procedure. Only < 1% of the unexplained variance in breakfast consumption was at the school level. Hence, adjustment for clustering effect was not done.

**Fig. 1:**
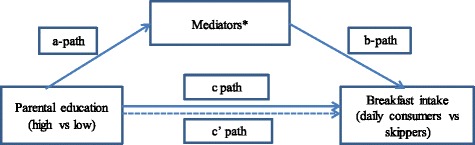
Mediation model. Legends: *The mediators included in the single mediation models were: parental modelling, parental co-participation in breakfast, parental rules, presence of breakfast foods and screen time (entered separately). The mediators included in the multiple mediation model were: parental modelling, presence of breakfast food and screen time (entered simultaneously). All paths were adjusted for gender and ethnicity

Descriptive analyses were first conducted. Thereafter, to explore the association between the included correlates and breakfast consumption and to explore mediating effects of these correlates in the association between parental education and breakfast consumption, mediation analyses were conducted [29]. Single mediation analyses were first conducted. In a single mediation, the a-path represents the association between parental education and the mediator. The b-path represents the association between the mediator and breakfast consumption adjusted for parental education. The c’ path represents the association between parental education and breakfast consumption (adjusted for the mediator). Significant mediators (screen time, parental modelling, perceived availability of breakfast food) were entered in the multiple mediation model. In the multiple mediation analysis, the a-paths represent the association between parental education and the mediators. The b-paths represent the association between the mediators and breakfast consumption (adjusted for parental education). The c’ path represents the association between education and breakfast consumption when adjusted for the mediators. Gender and ethnicity were adjusted for in all models. Figure 1 shows the mediation models followed. Bootstrap corrected CIs were calculated for indirect effects (a*b). Bootstrapping (1000 samples) was conducted using the PROCESS macro for SPSS by Andrew Hayes [29]. Figure 1 depicts the mediation model followed.

All analyses were conducted using IBM® SPSS® Statistics, version 22.0 (IBM Corp., Somers, New York, USA).

## Results

Participants with missing data on parental education (*n* = 36) were excluded from the analyses; the final sample therefore consisted of 702 participants. There was no statistically significant difference in gender, breakfast consumption and correlates of breakfast consumption between those excluded from the study due to missing data and those included. The sociodemographic characteristics of the adolescents are described in Table 1. The mean age was 13.6 (SD = 0.3), and 53% of participants were females. Only 9% of adolescents were of an ethnic minority background and 40% had parents with a low level of education. A total of 62% of adolescents were daily breakfast consumers.

**Table 1:** Description of the study sample (*n* = 706) and study variables

	Total sample	Boys (47%)	Girls (53%)	Breakfast skippers (48%)	Daily breakfast consumers (62%)
Age (yrs)	13.6 (0.3)	13.6 (0.3)	13.6 (0.3)	13.6 (0.3)	13.6 (0.3)
Parental education (% low education)	40	39	41	49	34
Ethnicity (% ethnic Norwegian)	9.2	9.6	8.9	11.0	8.2
Parental modelling^a^	4.6 (0.7)	4.6 (0.7)	4.6 (0.7)	4.4 (0.8)	4.8 (0.5)
Parental co-participation^a^	2.8 (2.2)	3.1 (2.3)	2.6 (2.1)	2.0 (1.7)	3.3 (2.2)
Availability of breakfast foods^a^	4.7 (0.6)	4.7 (0.6)	4.7 (0.5)	4.5 (0.7)	4.8 (0.5)
Rules regarding breakfast consumption^a^	3.8 (1.3)	3.6 (1.4)	3.8 (1.3)	3.4 (1.4)	3.9 (1.3)
Total screen time (hrs/day)	5.2 (2.5)	4.9 (2.5)	5.5 (2.5)	5.8 (2.6)	4.8 (2.4)

Results are presented as mean (CI) or as percentage, ^a^Range: 1–5

### Association between parental education and breakfast consumption (c-path)

There were significant differences in breakfast consumption between parental educational groups. Those who had parents with high education were significantly more likely to be daily breakfast consumers (OR = 1.97 (95% CI 1.43–2.72)) (Table 2).

**Table 2:** Correlates of breakfast consumption and their mediating effects in parental educational differences in breakfast consumption (*n* = 702)

	c-path (OR)	c’-path (OR)	a-path (B)	b-path (OR)	ab
Single mediation models					
Total screen time (hr/week)	**1.97 (1.43, 2.72)**	**1.69 (1.21, 2.36)**	**−1.134 (−1.511, −0.758)**	**0.85 (0.79, 0.91)**	**1.21 (1.11-1.35)**
Perceived modelling		**1.56 (1.12, 2.16)**	**0.254 (0.149, 0.358)**	**2.17 (1.70, 2.79)**	**1.22 (1.11, 1.39)**
Perceived availability		**1.73 (1.25, 2.40)**	**0.124 (0.033, 0.214)**	**2.21 (1.65, 2.97)**	**1.10 (1.03, 1.22)**
Perceived rules		**1.88 (1.36, 2.60)**	0.059 (−0.152, 0.269)	**1.36 (1.21, 1.53)**	1.02 (0.95, 1.09)
Perceived co-participation		**1.81 (1.30, 2.52)**	0.249 (−0.083, 0.582)	**1.37 (1.26, 1.49)**	1.08 (0.97, 1.21)
Multiple mediation model					
Total screen time (hr/week)	**1.97 (1.43, 2.72)**		**−1.143 (−1.520, −0.766)**	**0.84 (0.79, 0.91)**	**am**
Perceived modelling		1.35 (0.94, 1.93)	**0.275 (0.168, 0.381)**	**1.89 (1.45, 2.45)**	**1.19 (1.10, 1.35)**
Perceived availability			**0.137 (0.046, 0.228)**	**2.06 (1.51, 2.81)**	**1.10 (1.03, 1.22)**
Total					**1.59 (1.36, 1.97)**

Independent variable = parental education (ref = low) Dependent variable = breakfast consumption (ref = skippers)

The a-path represents the association between parental education and the mediator/s. The b-path represents the association between the mediator/s and breakfast consumption adjusted for parental education. The c’ path represents the association between parental education and breakfast consumption (adjusted for the mediator/s). The c path represents the unadjusted association between parental education and breakfast consumption. All paths were adjusted for gender and ethnicity

Bold values indicate statistically significant values

### Association between correlates and breakfast consumption (b-path, single mediation)

Higher levels of screen time were associated with lower odds of being a daily breakfast consumer (OR = 0.85 (95% CI 0.79, 0.91) for each hr/day increase in screen time).

A higher parental modelling (OR = 2.17 (95% CI 1.70, 2.79)), a higher availability of breakfast food (OR = 2.21 (95% CI 1.65, 2.97)), higher parental rules (OR = 1.36 (95% CI 1.21, 1.53)) and a higher parental co-participation (OR = 1.37 (95% CI 1.26, 1.49)) were associated with higher odds of being a breakfast consumer. These odds ratios represent the increase in odds of being a daily breakfast consumer for a unit increase in these variables (Table 2).

### Association between parental education and correlates of breakfast consumption (a path, single mediation)

There were significant differences between parental educational groups in parental modelling, with those with high parental education having parents who consume breakfast more often (*B* = 0.254 (95% CI 0.149, 0.358)). There were also small but significant differences in the availability of breakfast foods, the availability being higher among those with high parental education (*B* = 0.124 (95% CI 0.033, 0.214)). Total screen time (hrs/day) was significantly lower among those with high parental education (*B* = −1.134 (95% CI −1.511, −0.758)). There were no significant differences in perceived parental rules regarding breakfast consumption and in breakfast co-participation between parental educational groups (Table 2).

### Mediation effects of correlates in the association between parental education and breakfast consumption

On single mediation analyses, screen time, parental modelling and the availability of breakfast foods were found to be significant mediators of the association between parental education and breakfast consumption. No mediating effect of parental co-participation and parental rules related to breakfast consumption was found.

In the multiple mediation model, parental modelling, availability of breakfast food and screen time were found to be significant mediators of the association between parental education and breakfast consumption. The c’path (direct effect) became insignificant, indicating the presence of complete mediation (Table 2).

## Discussion

The study aimed to assess correlates of breakfast consumption among 13-year-old adolescents and to explore whether these correlates mediated the association between parental education and breakfast consumption. Parental modelling, parental co-participation, the availability of breakfast foods and parental rules related to breakfast consumption were positively related to daily breakfast consumption. Screen time was inversely related to daily breakfast consumption. Those with high parental education had significantly higher odds of being daily breakfast consumers. The association between parental education and breakfast consumption was mediated by parental modelling, the availability of breakfast foods and screen time.

The rate of breakfast consumption in this study is similar to that documented in a study including a nationally representative sample of Norwegian adolescents [1]. That study also found a small but significant decrease in breakfast consumption between 2002 and 2010, which is of particular concern and makes it particularly important to address breakfast consumption among adolescents.

Parental modelling was found to be positively associated with breakfast consumption, as previously documented in the literature [13, 14]. Parental co-participation in breakfast consumption was also found to be positively related to breakfast consumption. A previous study found that having breakfast with parents at age 10 years was related to more days of eating breakfast at age 16 years [16], indicating the important role parental co-participation in breakfast can play even on the long term. By being regular breakfast consumers and by doing so together with their children, parents can significantly positively influence the breakfast consumption behaviors of their children. Enforcing rules for breakfast consumption by parents also appears to play an important role, as previously found [12, 15]. Physical factors such as the availability of food have been consistently found to be positively related to different dietary behaviors among youth [30, 31], which was also the case for the availability of breakfast foods and the consumption of breakfast in this study. Another factor found to be significantly associated with breakfast consumption was screen time. Although the association between screen-based sedentary behaviors and the consumption of foods, in particular low nutrient high energy foods and drinks has been extensively explored [32, 33], the association with breakfast consumption has received less attention. The few existing studies have found an inverse association between breakfast consumption and television viewing [18] and screen time (TV and video game/computer) [19]; as well as inconclusive associations using both self-reported and objectively measured sedentary time [20]. The latter study however included non leisure-time screen-based sedentary activities. The inverse association between screen time and breakfast consumption found in this study could be related to different factors. As stated earlier, increased screen time has been found to be related to late sleeping [17]. This might result in waking up late which might be one of the reasons for skipping breakfast among adolescents. The potential mediating role of sleep in the association between screen time and breakfast consumption therefore needs to be assessed. Increased screen time can also be related to late snacking which might lead to a loss of appetite in the morning. There have been significant changes in the availability of different screens over the past decade. This can result in an increase in screen time, which might at least to some degree explain the documented small decrease in breakfast consumption over the past decade [1]. The association between screen time and breakfast consumption might however not necessarily be causal, but might be related to the so-called clustering of health behaviors [34]. Longitudinal studies and experimental studies are needed in the future to clarify this association.

As expected, parental educational differences in breakfast consumption were documented in this study. Correlates found to be related to parental education were screen time, parental modelling and the availability of breakfast foods. Screen time has been found to be inversely related to socioeconomic position in high income countries [35]. A systematic review of socioeconomic differences in correlates of dietary behaviors also found that modelling of dietary behaviors and availability of food were positively related to SEP, although these were not specific to breakfast consumption behaviour [36]. In line with our findings, the association between parental rules related to dietary behaviors and SEP was found to be generally null [36].

Parental modelling, the presence of breakfast foods and screen time were found to mediate parental educational differences in breakfast consumption; the association between breakfast consumption and parental education disappeared in the final model indicating complete mediation. To our knowledge, only two other studies have looked at mediators of socioeconomic differences in breakfast consumption and found attitudes [11] and parental modelling [25] to be significant mediators of socioeconomic differences in breakfast consumption. The studies however used different indicators of SEP than the present study (deprivation and maternal education) and identified only a single mediator. The present study adds to existing literature by showing that parental modelling, the presence of breakfast foods and screen time are important mediators of parental educational differences in breakfast consumption. Targeting these important modifiable correlates of breakfast consumption is likely to have a positive impact for all adolescents, but in particular for those with low parental education.

### Strengths and limitations

The results of this study should be seen in light of the following weaknesses. The cross-sectional data used in the study does not allow for any inference about causality. Ideally, mediation analyses should be conducted using longitudinal data. However, due to the nature of the variables included, the directions of associations in the mediation analyses in the present study are likely to be accurate (i.e. it is likely that parental education influenced the correlates and not vice versa). The use of self-report measures of dietary behaviours and correlates is another weakness of the present study as such measures are liable to recall and social desirability bias. However, there was evidence of reliability and validity of the measures used. The present study only included frequency of breakfast consumption; future studies looking at mediators of socioeconomic differences in breakfast content are needed. The strengths include the good participation rate in this study. The study also provides new information regarding mediators of socioeconomic differences in breakfast consumption. Parental education was reported by parents themselves which was a strength as it allowed for fairly complete data; parental reports of education are also likely to be more reliable than adolescent reports. However, education was the only indicator of SEP included. Parental education and other indicators of SEP such as income and occupation are correlated. However, existing research suggests that the association between these indicators of SEP and dietary behaviours among children can be specific/independent (37). Therefore, including measures of income and occupation in future studies might allow for the exploration of the independent effects of these indicators on breakfast consumption. Finally, computing total screen time from different screen-based activities might result in an overestimation of time spent on screen-based activities as these activities can co-occur. However, this would more likely affect the strength of associations and to a lesser extent the directions of associations.

## Conclusions

The rate of breakfast skipping found in this study was high and was more pronounced among those with low parental education. Increasing the availability of breakfast food, improving parental modelling of breakfast consumption and targeting screen time appear to be promising strategies to reduce parental educational differences in breakfast consumption.

## Additional file

Additional file 1: Questionnaire items. (DOCX 18 kb)

## Abbreviations

ESSENS: Environmental determinantS of dietary behaviorS among adolescENtS
SEP: Socioeconomic position
