# Supplementary material for: Mediators of the association between parental education and breakfast consumption among adolescents : the ESSENS study
Source: BMC Pediatr. 2017 Feb 23;17:61. doi: 10.1186/s12887-017-0811-2 (PMC5322630; doi:10.1186/s12887-017-0811-2)
Supplement: Additional file 1: — Questionnaire items. (DOCX 18 kb) [file 12887_2017_811_MOESM1_ESM.docx]

Questionnaire items

Sociodemographic characteristics

Are you a girl or a boy?

Girl

Boy

In which year are you born?

2000

2001

2002

2003

Another year ---------------------------

In which month are you born?

 January

 February

 March

 April

 May

 June

 July

 August

 September

 October

 November

 December

In which country is your mother born?

Norway

Another country

In which country is your father born?

Norway

Another country

Breakfast

(When we say breakfast we mean the first things you usually eat and drink within 2 hours after getting up in the morning. This can be at home, on the way to school or just before entering school. During weekends breakfast is anything you drink and/or eat before 11 a.m.)

On a regular school week from Monday to Friday, on how many days do you usually eat breakfast?

I never eat breakfast on schooldays

1 day

2 days

3 days

4 days

5 days

How often do you eat breakfast on weekend days (Saturday and Sunday)?

I never eat breakfast on weekend days

I usually eat breakfast on 1 weekend day (Saturday OR Sunday)

I usually eat breakfast on both weekend days (Saturday AND Sunday)

How often do your parents/caregivers eat breakfast?

Always

Often

Sometimes

Not often

Never

How often do you eat breakfast with your parents/caregivers?

Never

Less than once a week

Once a week

2-4 days a week

5-6 days a week

Every day

Are there usually breakfast products (milk, cereals, bread etc) at your home?

Always

Often

Sometimes

Not often

Never

My parents/caregivers have rules about whether I should eat breakfast

I fully agree

I agree a bit

Neither agree nor disagree

I disagree a bit

I fully disagree

Sedentary time

About how many hours a day do you usually watch television in your free time? (include DVD, video or film on a computer, telephone or ipad)

Mark one box for weekdays and one box for weekend days

Weekdays (average of all weekdays)

 None at all

 Less than 30 minutes/day

 1,0 hours/day

 1,5 hours/day

 2,0 hours/day

 2,5 hours/day

 3,0 hours/day

 3,5 hours/day

 4,0 hours/day or more

Weekend day (average of a Saturday or Sunday)

 None at all

 Less than 30 minutes/day

 1,0 hours/day

 1,5 hours/day

 2,0 hours/day

 2,5 hours/day

 3,0 hours/day

 3,5 hours/day

 4,0 hours/day or more

About how many hours per day do you usually play computer games, game consoles (Playstation, Xbox, GameCube), game on ipad or game on mobile phone?

Weekdays (average of all weekdays)

 None at all

 Less than 30 minutes/day

 1,0 hours/day

 1,5 hours/day

 2,0 hours/day

 2,5 hours/day

 3,0 hours/day

 3,5 hours/day

 4,0 hours/day or more

Weekend day (average of a Saturday or Sunday)

 None at all

 Less than 30 minutes/day

 1,0 hours/day

 1,5 hours/day

 2,0 hours/day

 2,5 hours/day

 3,0 hours/day

 3,5 hours/day

 4,0 hours/day or more

55. About how many hours per day do you use computer, ipad or mobile phone for activities such as chatting, email, internet, Facebook, Instagram in your free time?

Mark one box for weekdays and one box for weekend days

Weekdays (average of all weekdays)

 None at all

 Less than 30 minutes/day

 1,0 hours/day

 1,5 hours/day

 2,0 hours/day

 2,5 hours/day

 3,0 hours/day

 3,5 hours/day

 4,0 hours/day or more

Weekend day (average of a Saturday or Sunday)

 None at all

 Less than 30 minutes/day

 1,0 hours/day

 1,5 hours/day

 2,0 hours/day

 2,5 hours/day

 3,0 hours/day

 3,5 hours/day

 4,0 hours/day or more
